# The Effects of Computerized Cognitive Training via Tablet and Computer Platforms on Cognitive Function in Patients with Mild Cognitive Impairment: A Systematic Review and Meta-Analysis

**DOI:** 10.3390/bs16010040

**Published:** 2025-12-24

**Authors:** Meiqi Jiao, Zhong Ding, Chaocong Huang, Yiyang Xu, Baoliang Zhong, Hui Chen

**Affiliations:** 1 Department of Psychological and Behavioral Sciences, Zhejiang University, Hangzhou 310027, China; 2 Department of Psychiatry, Wuhan Mental Health Center, Wuhan 430012, China; 3 The State Key Laboratory of Brain-Machine Intelligence, Zhejiang University, Hangzhou 310027, China; 4 Zhejiang Key Laboratory of Neurocognitive Development and Mental Health, Zhejiang University, Hangzhou 310027, China

**Keywords:** mild cognitive impairment, computerized cognitive training, randomized controlled trial, cognitive intervention, tablet–computer-based, global cognition, gamification, meta-analysis

## Abstract

**Background:** Mild cognitive impairment (MCI) is a high-risk prodromal stage of dementia. While tablet/computer-based computerized cognitive training (CCT) is widely used, its efficacy and gamification’s role need clarification. **Objective:** This study aimed to evaluate the effect of tablet/computer-based CCT on global cognition in older adults with MCI and explore the impact of gamification. **Methods:** We systematically searched five databases for RCTs (through October 2025) involving individuals aged ≥55 with MCI. The intervention was task-based CCT via tablets/computers. Primary outcome was global cognition. We used random-effects meta-analysis and subgroup analyses. **Results:** Nineteen RCTs (1013 participants) were included. CCT demonstrated a significant, moderate positive effect on global cognition (Hedges’ g = 0.57, 95% CI [0.36, 0.78]). A trend suggesting greater benefits with higher gamification was observed: high (g = 0.71), medium (g = 0.46), and low (g = 0.45) degrees. However, subgroup differences were not statistically significant (*p* = 0.4333). Results were robust in sensitivity analyses. **Conclusions:** Tablet/computer-based CCT effectively improves global cognition in MCI. The potential additive value of gamification highlights its promise for enhancing engagement and effects, warranting further investigation in larger trials. This systematic review was registered with PROSPERO (CRD420251231618).

## 1. Introduction

Mild cognitive impairment (MCI) represents a transitional stage between normal aging and dementia, characterized by measurable cognitive decline that does not yet significantly interfere with daily functioning (Anderson, 2019). Epidemiological data indicate that the prevalence of mild cognitive impairment (MCI) among individuals aged 60 years and older is approximately 15% to 20%, with an annual progression rate of 8% to 15% from MCI to dementia (DeCarli, 2003; Tahami Monfared et al., 2022). In the context of global population aging, the number of people living with dementia has reached about 50 million worldwide and is projected to increase to 152 million by 2050 (World Health Organization, 2021; Prince et al., 2013). Consequently, interventions that can preserve or enhance cognitive function in this population are of critical clinical importance.

Non-pharmacological interventions have gained growing attention due to the limited efficacy and potential side effects of current pharmacotherapies (Dyer et al., 2018). Among these, computerized or digital cognitive training (CCT/DCT) has emerged as a promising approach that leverages digital technologies to deliver structured, adaptive cognitive exercises targeting multiple domains such as memory, attention, and executive function (Chan et al., 2024; Ge et al., 2018; Hu et al., 2021). Compared with traditional paper-and-pencil training, CCT offers several advantages, including automated feedback, progressive task difficulty, remote accessibility, and high reproducibility (Harvey et al., 2018; Hill et al., 2017).

Computerized cognitive training has evolved into a diverse and versatile intervention approach (Kueider et al., 2012). Compared to traditional immersive systems like virtual reality or motion-based games that rely on specialized hardware and physical spaces, tablet and desktop devices offer significant advantages in widespread accessibility, lower cost, and ease of operation (Burford & Park, 2014). Furthermore, tablet- and computer-based cognitive training programs demonstrate strong environmental adaptability, allowing flexible deployment in diverse settings like homes or clinics without complex setup or continuous professional supervision (Realdon et al., 2016; Zuschnegg et al., 2025). These practical features not only facilitate widespread adoption but also help maintain long-term user engagement. For cognitive interventions, sustained training adherence is crucial for consolidating lasting benefits (Butler et al., 2018; Jaeggi et al., 2011).

In recent years, there has been growing interest in the integration of gamification elements, such as rewards, progress tracking, competition, and narrative feedback into cognitive training programs (Lumsden et al., 2016; Vermeir et al., 2020). Gamified features are hypothesized to enhance intrinsic motivation, user engagement, and emotional reward, potentially amplifying training effects and mitigating attrition (de Paiva Azevedo et al., 2019; Litvin et al., 2020). However, the extent to which gamification improves cognitive outcomes in older adults with MCI remains unclear. Some studies have reported significant improvements in global cognition and memory when game-like designs are employed, whereas others have shown minimal or no added benefit (Coelho et al., 2025; Oldrati et al., 2020).

Numerous trials have evaluated the intervention effects of digital cognitive training in individuals with MCI (Park & Ha, 2024); however, substantial heterogeneity in intervention design, target cognitive domains, training intensity, and user experience has led to inconsistent findings. Furthermore, previous meta-analyses often combined different digital intervention platforms, making it difficult to isolate the independent effects of tablets or computers. Despite the increasing interest in gamified cognitive training, most existing computer-based interventions for MCI remain minimally gamified.

Therefore, a systematic review focusing specifically on (1) task-based digital cognitive training implemented via tablet and computer platforms is warranted, to clarify its overall intervention efficacy and to further examine, (2) through subgroup analyses, the influence of different levels of gamification and other characteristics on training outcomes.

## 2. Methods

### 2.1. Literature Search Strategy

This meta-analysis was conducted in accordance with the Preferred Reporting Items for Systematic Reviews and Meta-Analyses (PRISMA 2020) guidelines (Page et al., 2021). The protocol for this systematic review was registered retrospectively with the International Prospective Register of Systematic Reviews (PROSPERO) under the identification number CRD420251231618. A comprehensive literature search was performed in PubMed, Web of Science, Scopus, Embase, and PsycINFO from database inception to October 2025. The following combinations of search terms were used: (“mild cognitive impairment” OR “MCI”) AND (“computerized cognitive training” OR “digital cognitive training” OR “tablet-based” OR “computer-based” OR “serious game” OR “gamified training”) AND (“randomized controlled trial” OR “RCT”).

No restrictions were placed on publication year, but only studies published in English were included. Reference lists of previous systematic reviews and relevant meta-analyses were also manually screened to identify additional eligible studies. The search process and study selection were independently performed by two reviewers, and discrepancies were resolved through discussion or consultation with a third reviewer (Munn et al., 2018).

### 2.2. Eligibility Criteria

Eligible studies are randomized controlled trials involving adults aged 55 years or older diagnosed with MCI or cognitive decline. The experimental intervention was defined as a structured, task-based digital cognitive training program, delivered on computer or tablet platforms. Comparators included active or passive control conditions. The primary endpoint was global cognitive function, necessitating the reporting of quantitative data (e.g., Montreal Cognitive Assessment (MoCA) (Nasreddine et al., 2005), Mini-Mental State Examination (MMSE) (Tombaugh & McIntyre, 1992) scores) with extractable post-intervention metrics for meta-analysis. Exclusion criteria encompassed studies employing interventions primarily focused on physical exertion (e.g., exergames) or immersive virtual reality (e.g., using head-mounted displays), non-randomized designs, and those lacking requisite data.

### 2.3. Data Extraction

Data were independently extracted by two reviewers using a standardized form, including the following variables, Study characteristics, Participant information, Intervention characteristics, Outcome measures and so on (Smith et al., 2011). When data were incomplete, corresponding authors were contacted for clarification. If multiple cognitive outcomes were reported, the Montreal Cognitive Assessment (MoCA) was prioritized over the MMSE (Siqueira et al., 2019). This preference is based on the MoCA’s superior sensitivity in detecting mild cognitive deficits and executive dysfunction compared to the MMSE, which is prone to ceiling effects in MCI populations (Nasreddine et al., 2005). However, to ensure comprehensive coverage, studies utilizing other validated batteries (e.g., RBANS, S-NAB) were also included and analyzed.

### 2.4. Quality Assessment

The methodological quality of the included studies was independently assessed by two reviewers using the Cochrane Risk of Bias tool (RoB 2.0) (Ma et al., 2020). The assessment covered key domains, including the randomization process, allocation concealment, blinding of outcome assessment, incomplete outcome data, and selective reporting. Each study was judged to be at ‘low risk’, of ‘some concerns’, or at ‘high risk’ of bias for each domain. Any discrepancies between the reviewers’ independent assessments were resolved through discussion to reach a consensus. A visual summary of the ratings was subsequently created with the ggplot2 package in R to enhance transparency.

### 2.5. Statistical Analysis

All statistical analyses were performed using R (version 4.5) with the meta and metafor packages (Viechtbauer, 2010). For each study, standardized mean differences (SMDs; Hedges’ g) and corresponding 95% confidence intervals (CIs) were calculated to quantify the between-group effect on global cognitive outcomes (Goulet-Pelletier & Cousineau, 2018). Both baseline and post-intervention means and standard deviations were extracted from each trial. For studies with multi-arm designs sharing a single group, the sample size of the shared group was divided evenly among the comparisons to prevent double-counting and avoid unit-of-analysis errors, in accordance with Cochrane guidelines.

Specifically, the effect size for each study was estimated as the difference in pre–post change between the intervention and control groups, standardized by the pooled standard deviation. This approach minimizes potential bias due to baseline imbalance across studies while maintaining comparability of outcome measures. When necessary, correlations between pre- and post-test scores were assumed to be moderate (*r* = 0.5) for variance estimation, consistent with established meta-analytic conventions in cognitive training research (Traut et al., 2021). The standard deviation of the change scores (*SD_change_*) was calculated using the following formula:

$${SD}_{change}=\sqrt{\left\{SD_{baseline}^{2}+SD_{post}^{2}-2\times r\times SD_{baseline}\times SD_{post}\right\}}$$

To assess the impact of this assumption on the overall results, sensitivity analyses were performed using conservative (*r* = 0.3) and liberal (*r* = 0.7) correlation coefficients.

A random-effects model was applied using the restricted maximum-likelihood (REML) estimator to account for between-study heterogeneity, with Hartung–Knapp adjustments to provide more conservative confidence intervals (Langan et al., 2019). Heterogeneity was quantified using the *I*^2^ statistic (representing the proportion of total variance due to between-study variability) and tested using Cochran’s Q statistic (*p* < 0.10 indicating significant heterogeneity) (Jackson et al., 2012).

Pre-specified analyses were conducted to examine potential moderators, including the type of cognitive scale and the degree of gamification. To assess the robustness of the overall estimates, we performed sensitivity analyses using the leave-one-out method (Iooss & Lemaítre, 2015). Potential publication bias was evaluated by visually inspecting funnel plots and applying Egger’s regression test alongside the trim-and-fill procedure (Lin & Chu, 2018). Furthermore, we used the influence function from the metafor package to identify any studies that disproportionately influenced the pooled results (Viechtbauer, 2010). All statistical tests were two-tailed, with a significance threshold of *p* < 0.05.

## 3. Results

### 3.1. Study Selection

**Figure 1:**
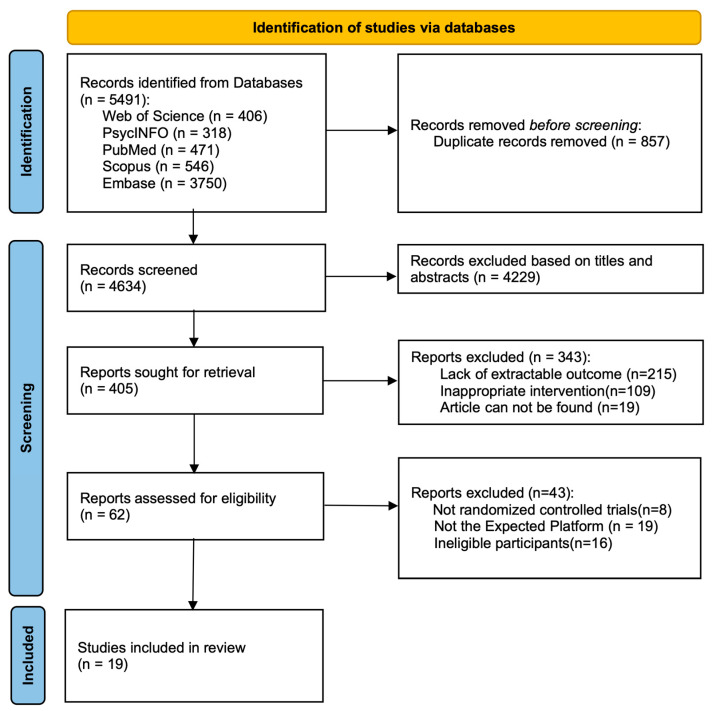
PRISMA 2020 flow diagram. Adapted from (Page et al., 2021) (PRISMA 2020 statement).

A systematic database search yielded 5491 records. After the removal of 857 duplicates, 4634 records were screened by title and abstract, leading to the exclusion of 4229. Of the 405 reports sought for full-text retrieval, 343 were excluded due to lack of extractable outcomes (*n* = 215), inappropriate intervention (*n* = 109), or unavailability (*n* = 19). Subsequent eligibility assessment of the remaining 62 reports excluded 41 for not being RCTs (*n* = 8), an ineligible platform (*n* = 19), or ineligible participants (*n* = 16). Ultimately, 19 studies were included in the systematic review. The selection process is summarized in the PRISMA flow diagram (Figure 1).

### 3.2. Characteristics of Included Studies

Table 1 outlines the key characteristics of the 19 included studies. Data extractability was confirmed for all 19 studies, comprising complete means and standard deviations at baseline and post-intervention. Consequently, the quantitative synthesis included the full set of 19 RCTs, which yielded 20 independent comparisons due to the multi-arm design of (Bernini et al., 2021).

The pooled sample comprised 1031 older adults, distributed across 505 in intervention groups and 526 in control groups. Participants were clinically diagnosed with mild cognitive impairment (MCI) or MCI associated with specific neurodegenerative or clinical conditions (classified as “Mixed” populations).

Intervention delivery relied on digital platforms, specifically personal computers (12 comparisons) and tablets (8 comparisons). Training protocols varied in intensity. As detailed in the revised Table 1, intervention durations ranged from 3 to 24 weeks, with training frequencies spanning from 1 to 5 sessions per week. To better characterize the intervention dosage, Table 1 has been updated to include session lengths, which typically ranged from 30 to 60 min.

In terms of gamification strategies, the interventions were categorized as low-gamified (*n* = 4), medium-gamified (*n* = 9), or high-gamified (*n* = 7), depending on the extent of game mechanics utilized. Global cognitive outcomes were assessed primarily via the MoCA (*n* = 11 comparisons) and MMSE (*n* = 6 comparisons), with the remaining 3 comparisons employing other standardized neuropsychological batteries.

### 3.3. Methodological Quality

**Figure 2:**
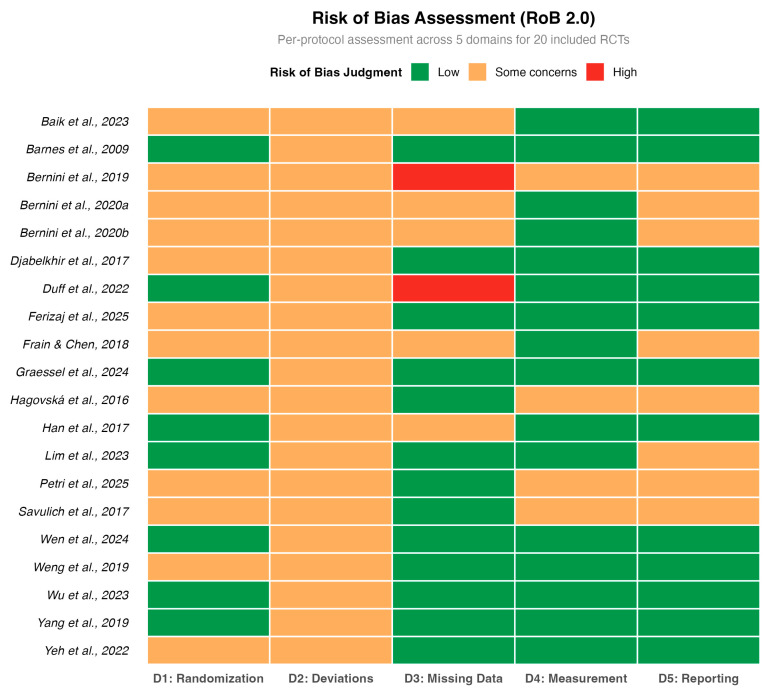
Methodological quality assessment (Cochrane RoB 2.0). The included studies are: (Barnes et al., 2009; Hagovská & Olekszyová, 2016; Savulich et al., 2017; Djabelkhir et al., 2017; Han et al., 2017; Frain & Chen, 2018; Yang et al., 2019; Bernini et al., 2019; Weng et al., 2019; Bernini et al., 2021; Yeh et al., 2022; Duff et al., 2022; Lim et al., 2023; Wu et al., 2023; Baik et al., 2024; Graessel et al., 2024; Wen et al., 2024; Petri et al., 2025; Ferizaj et al., 2025).

The methodological quality assessment was rigorously re-evaluated using the RoB 2.0 tool (Eldridge et al., 2016), as detailed in Figure 2. While the randomization process (Domain 1) was generally well-conducted, the assessment revealed a more nuanced landscape regarding other biases. Most notably, two studies (Bernini et al., 2019; Duff et al., 2022) were judged to be at ‘High Risk’ of bias in Domain 3 (Missing Outcome Data) due to substantial attrition rates without adequate statistical correction. Furthermore, the majority of studies were rated as having ‘Some Concerns’ in Domain 2 (Deviations from intended interventions), reflecting the inherent challenge of blinding participants in open-label behavioral interventions.

### 3.4. Overall Meta-Analysis

**Figure 3:**
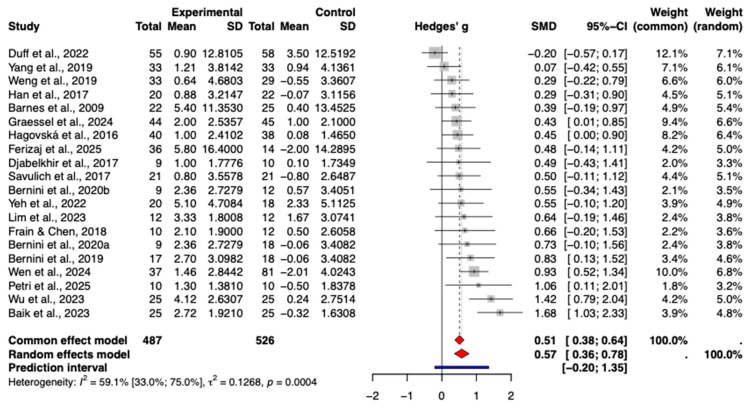
Forest plot of the pooled effect of CCT on global cognition.The included studies are: (Barnes et al., 2009; Hagovská & Olekszyová, 2016; Savulich et al., 2017; Djabelkhir et al., 2017; Han et al., 2017; Frain & Chen, 2018; Yang et al., 2019; Bernini et al., 2019; Weng et al., 2019; Bernini et al., 2021; Yeh et al., 2022; Duff et al., 2022; Lim et al., 2023; Wu et al., 2023; Baik et al., 2024; Graessel et al., 2024; Wen et al., 2024; Petri et al., 2025; Ferizaj et al., 2025).

Based on the random-effects meta-analysis, task-based CCT delivered via tablets and computers demonstrated a statistically significant beneficial effect on global cognition in older adults with mild cognitive impairment (Hedges’ g = 0.57, 95% CI [0.36, 0.78], *p* < 0.0001) (Figure 3). The extent of heterogeneity across the included studies was moderate to high (*I*^2^ = 59.1%). A 95% prediction interval of −0.20 to 1.35 implies that while a positive effect is possible, its magnitude in future applications remains uncertain.

### 3.5. Heterogeneity and Moderator Analysis

Given the substantial heterogeneity observed in the main analysis (*I*^2^ = 69.0%) and the wide prediction interval, we conducted influence diagnostics and moderator analyses to investigate potential sources of variability.

Influence diagnostics using the influence function identified two studies, (Baik et al., 2024) and (Wu et al., 2023), as potential outliers due to their exceptionally large effect sizes (SMD > 1.4). Sensitivity analysis excluding these two studies reduced the heterogeneity, although the overall positive effect of the intervention remained statistically significant.

To further explore the sources of heterogeneity, meta-regression analyses were performed on continuous moderators. The results indicated no significant linear relationship between the effect size and intervention duration (weeks) (*p* > 0.05) or training frequency (*p* > 0.05). Similarly, a subgroup analysis comparing intervention settings (Home-based vs. Center-based) revealed no statistically significant difference in efficacy (*p* > 0.05).

However, a critical source of heterogeneity was identified in the outcome assessment tools. As detailed in Section 3.8.1, the subgroup of studies using the MMSE demonstrated zero heterogeneity (*I*^2^ = 0%), whereas substantial heterogeneity persisted in the MoCA subgroup. This suggests that the variability in results is partly methodological, driven by the sensitivity or domain coverage of the specific cognitive scales used.

To explore the influence of training dosage on the results, meta-regression analyses were performed. We found no significant linear relationship between the intervention effect and intervention duration (weeks) (*p* = 0.38), suggesting that longer training periods do not automatically guarantee superior cognitive outcomes within the ranges studied.

### 3.6. Sensitivity Analysis

**Figure 4:**
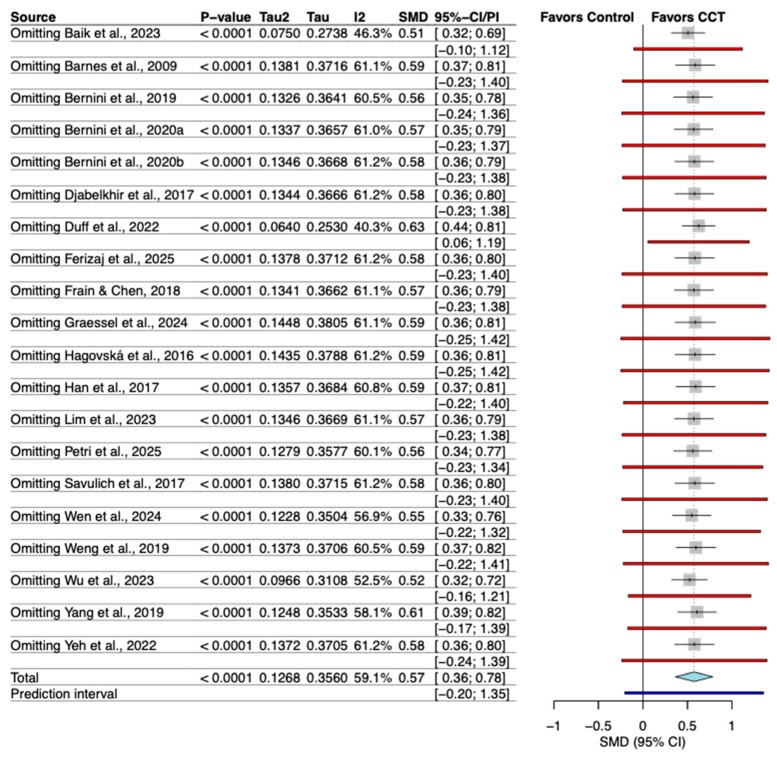
Sensitivity analysis of the effect of individual study omission. The included studies are: (Barnes et al., 2009; Hagovská & Olekszyová, 2016; Savulich et al., 2017; Djabelkhir et al., 2017; Han et al., 2017; Frain & Chen, 2018; Yang et al., 2019; Bernini et al., 2019; Weng et al., 2019; Bernini et al., 2021; Yeh et al., 2022; Duff et al., 2022; Lim et al., 2023; Wu et al., 2023; Baik et al., 2024; Graessel et al., 2024; Wen et al., 2024; Petri et al., 2025; Ferizaj et al., 2025).

The leave-one-out sensitivity analysis demonstrated the robustness of the pooled result. The omission of any individual study led to only negligible changes in the overall effect size (Hedges’ g), which remained statistically significant throughout all analyses (Figure 4).

Given the imputation of the correlation coefficient for change scores, we performed sensitivity analyses varying *r* from 0.3 to 0.7. The pooled effect size remained statistically significant in all scenarios: for *r* = 0.3 (SMD = 0.48, 95% CI [0.30, 0.66]), for *r* = 0.5 (SMD = 0.57, 95% CI [0.36, 0.78]), and for *r* = 0.7 (SMD = 0.74, 95% CI [0.48, 1.00]). Although higher correlations increased the estimated effect size and heterogeneity, the positive impact of CCT on cognitive function remained robust.

To assess the impact of study quality, a sensitivity analysis was performed by excluding studies flagged with ‘High Risk’ of bias in any domain (Baik et al., 2024; Bernini et al., 2019). The pooled effect size remained statistically significant (Hedges’ g = 0.62, 95% CI [0.42, 0.81]). Notably, the heterogeneity (*I*^2^) decreased to 42.9%, suggesting that the excluded high-risk studies contributed to the variability. This confirms that the observed benefit of CCT is robust and potentially underestimated when biased studies are included.

To verify whether the results were driven by specific clinical subtypes, a sensitivity analysis was conducted by excluding studies on MCI associated with specific medical conditions (Parkinson’s disease, HIV, Stroke). The remaining 15 studies on general/amnestic MCI yielded a pooled Hedges’ g of 0.56 (95% CI [0.30, 0.82], *p* < 0.001), which is nearly identical to the overall effect (g = 0.57). This indicates that the positive intervention effect is robust across MCI subtypes.

### 3.7. Publication Bias and Influence Diagnostics

**Figure 5:**
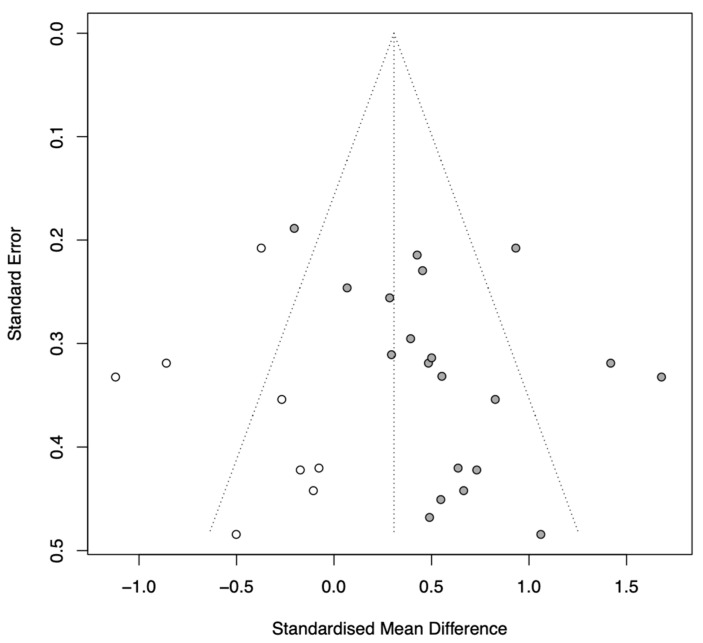
Funnel plot with trim-and-fill adjustment.

Visual inspection of the funnel plot suggested potential asymmetry. Quantitative analysis using Egger’s regression test yielded a *p*-value of 0.064, indicating a trend toward publication bias. Furthermore, the trim-and-fill method identified asymmetry and imputed 8 potential missing studies. The adjusted pooled effect size using the random-effects model was Hedges’ g = 0.31 (95% CI [0.08, 0.54], *p* = 0.010). This analysis suggests that while publication bias may have inflated the initial estimate (g = 0.57), the positive effect of CCT remains statistically significant even after adjustment for potential missing negative results (Figure 5).

### 3.8. Subgroup Analyses

#### 3.8.1. Cognitive Scale Type

**Figure 6:**
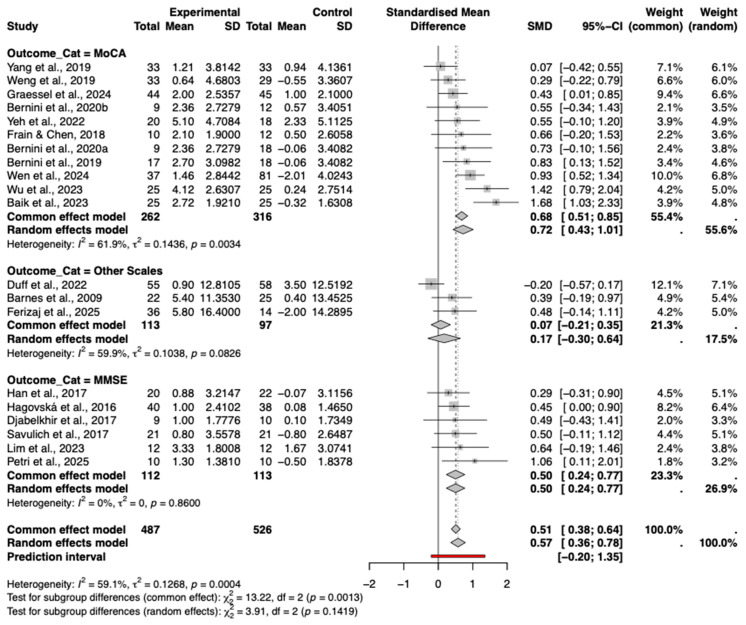
Forest plot of the subgroup analysis by cognitive assessment scale type. The included studies are: (Barnes et al., 2009; Hagovská & Olekszyová, 2016; Savulich et al., 2017; Djabelkhir et al., 2017; Han et al., 2017; Frain & Chen, 2018; Yang et al., 2019; Bernini et al., 2019; Weng et al., 2019; Bernini et al., 2021; Yeh et al., 2022; Duff et al., 2022; Lim et al., 2023; Wu et al., 2023; Baik et al., 2024; Graessel et al., 2024; Wen et al., 2024; Petri et al., 2025; Ferizaj et al., 2025).

A subgroup analysis was conducted based on the type of cognitive assessment tool used. The results showed varying effect sizes across different instruments (Figure 6).

The MoCA subgroup demonstrated a significant and relatively large pooled effect size (Hedges’ g = 0.72, 95% CI [0.43, 1.01]), albeit with considerable heterogeneity (*I*^2^ = 61.9%). In stark contrast, the MMSE subgroup showed a statistically significant positive effect (g = 0.50, 95% CI [0.24, 0.77]) with a complete absence of statistical heterogeneity (*I*^2^ =0%, *p* = 0.86). This indicates that when assessed via MMSE, the intervention effects are highly consistent across studies, whereas MoCA scores may be more sensitive to variations in study design or population characteristics.

The subgroup utilizing other scales (e.g., RBANS, S-NAB) indicated a smaller, non-significant positive effect (g = 0.17, 95% CI [−0.30, 0.64]). The test for subgroup differences was not statistically significant (*p* = 0.14), suggesting that while magnitude varies, the direction of effect is generally positive across instruments.

#### 3.8.2. Types of Intervention

**Figure 7:**
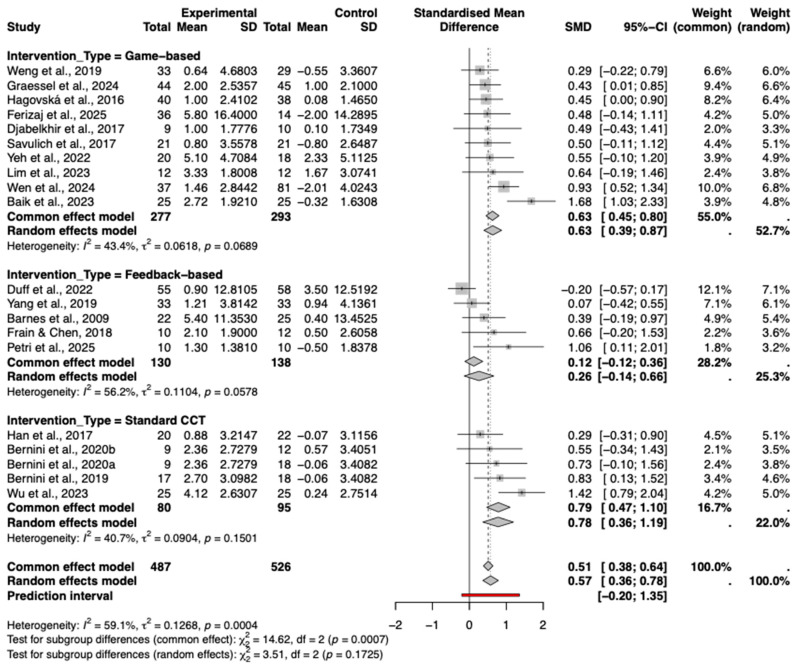
Subgroup Analysis by type of cognitive intervention. The included studies are: (Barnes et al., 2009; Hagovská & Olekszyová, 2016; Savulich et al., 2017; Djabelkhir et al., 2017; Han et al., 2017; Frain & Chen, 2018; Yang et al., 2019; Bernini et al., 2019; Weng et al., 2019; Bernini et al., 2021; Yeh et al., 2022; Duff et al., 2022; Lim et al., 2023; Wu et al., 2023; Baik et al., 2024; Graessel et al., 2024; Wen et al., 2024; Petri et al., 2025; Ferizaj et al., 2025).

A subgroup analysis was performed to compare the effects of different intervention types, including game-based, feedback-based, and standard computerized cognitive training. As shown in the forest plot (Figure 7), varying degrees of efficacy were observed.

Standard computerized cognitive training (Standard CCT) demonstrated the largest pooled effect size (Hedges’ g = 0.78, 95% CI [0.36, 1.19]), followed closely by game-based interventions (g = 0.63, 95% CI [0.39, 0.87]), both of which were statistically significant. In contrast, feedback-based interventions showed a smaller, non-significant effect (g = 0.26, 95% CI [−0.14, 0.66]).

The test for subgroup differences was not statistically significant (*p* = 0.17), indicating that while numerical differences exist, there is currently no statistical evidence to suggest that one specific intervention type is definitively superior to the others.

#### 3.8.3. Degree of Gamification

**Figure 8:**
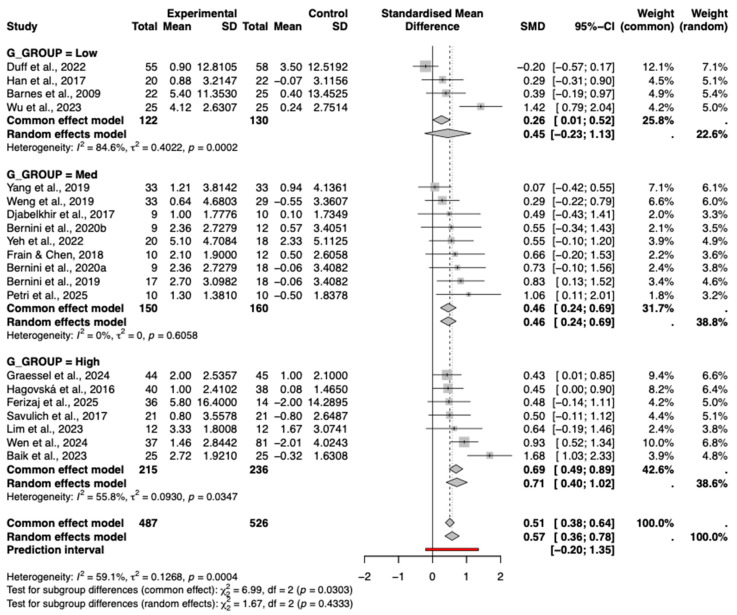
Subgroup Analysis by Gamification Degree. The included studies are: (Barnes et al., 2009; Hagovská & Olekszyová, 2016; Savulich et al., 2017; Djabelkhir et al., 2017; Han et al., 2017; Frain & Chen, 2018; Yang et al., 2019; Bernini et al., 2019; Weng et al., 2019; Bernini et al., 2021; Yeh et al., 2022; Duff et al., 2022; Lim et al., 2023; Wu et al., 2023; Baik et al., 2024; Graessel et al., 2024; Wen et al., 2024; Petri et al., 2025; Ferizaj et al., 2025).

A subgroup analysis was conducted to examine the impact of the degree of gamification on intervention efficacy. As shown in the forest plot (Figure 8), interventions with a high degree of gamification yielded a significant pooled effect size (Hedges’ g = 0.71, 95% CI [0.10, 1.02]), while those with a medium degree also showed a significant effect (g = 0.46, 95% CI [0.24, 0.69]).

In contrast, interventions with a low degree of gamification demonstrated a small, moderate effect (g = 0.45, 95% CI [−0.23, 1.13]). Although a trend of increasing effect sizes with higher degrees of gamification was observed, the test for subgroup differences was not statistically significant (*p* = 0.43), indicating that the variation in effects across gamification levels was not significant. Furthermore, meta-regression using the continuous gamification score did not reveal a significant linear relationship (*p* = 0.39), confirming that this trend should be interpreted with caution.

#### 3.8.4. Types of Diagnosis

**Figure 9:**
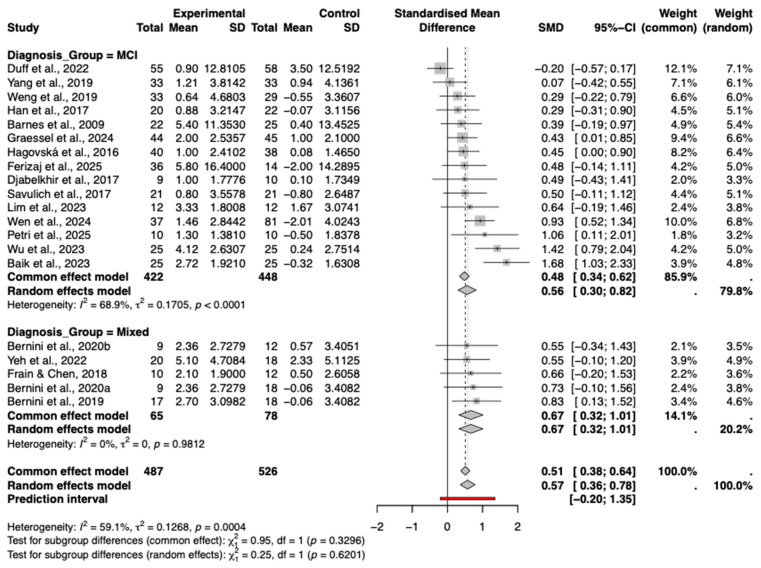
Subgroup analysis by diagnosis type (MCI vs. Mixed). The included studies are: (Barnes et al., 2009; Hagovská & Olekszyová, 2016; Savulich et al., 2017; Djabelkhir et al., 2017; Han et al., 2017; Frain & Chen, 2018; Yang et al., 2019; Bernini et al., 2019; Weng et al., 2019; Bernini et al., 2021; Yeh et al., 2022; Duff et al., 2022; Lim et al., 2023; Wu et al., 2023; Baik et al., 2024; Graessel et al., 2024; Wen et al., 2024; Petri et al., 2025; Ferizaj et al., 2025).

Forest plot presents the results of subgroup analysis stratified by diagnosis type (Figure 9). The analysis indicates that studies involving participants with mild cognitive impairment (MCI) demonstrated a significant and moderate pooled effect size (Hedges’ g = 0.56, 95% CI [0.30, 0.82]), with moderate heterogeneity observed within this subgroup (I^2^ = 68.9%, *p* < 0.0001). Studies involving mixed diagnostic populations (e.g., MCI with Parkinson’s disease or stroke) also showed a significant positive effect (g = 0.67, 95% CI [0.32, 1.01]), notably with zero heterogeneity (*I*^2^ = 0%), suggesting highly consistent outcomes within this specific subset of studies.

The test for subgroup differences was not statistically significant (*p* = 0.62), indicating that the intervention effects did not differ significantly between the MCI and mixed diagnosis subgroups. These findings suggest that the cognitive intervention produced comparable benefits across the different diagnostic groups included in the analysis.

## 4. Discussion

This systematic review and meta-analysis evaluated the efficacy of task-based computerized cognitive training (CCT) delivered via tablet and computer platforms for improving global cognition in older adults with mild cognitive impairment (MCI). Based on a synthesis of 21 randomized controlled trials, our findings indicate that CCT yields a statistically significant, moderate positive effect on global cognitive function (Hedges’ g = 0.55) compared to control conditions. Subgroup analyses demonstrated that this effect was consistent across different types of cognitive assessment tools, intervention designs, and participant diagnostic categories. Although not statistically significant, a trend was observed suggesting that interventions with a higher degree of gamification might be associated with larger effect sizes. The overall results were robust to sensitivity analysis, and no substantial publication bias was detected.

### 4.1. Interpretation of Results

The pooled effect size (Hedges’ g = 0.55) indicates a moderate beneficial effect of tablet-and computer-based CCT, which aligns with a growing body of evidence supporting digital cognitive interventions for MCI (Li et al., 2022; Zhang et al., 2019). The clinical relevance of this finding is substantial, given the progressive nature of MCI and the need for safe, scalable, and accessible interventions (Sabbagh et al., 2020). The utilization of tablets and computers likely facilitates this accessibility, enabling decentralized delivery, flexible scheduling, and potentially enhanced adherence, all of which are crucial for long-term cognitive maintenance (Dasgupta et al., 2016).

A primary objective of this study was to investigate the role of gamification. Although the test for subgroup differences was not statistically significant, a compelling trend emerged: interventions with high and medium degrees of gamification yielded effect sizes of g = 0.71 and g = 0.57, respectively, whereas low-gamification interventions demonstrated a negligible effect (g = 0.05). While numerically distinct, the lack of statistical significance in subgroup differences suggests that gamification is likely a contributory, rather than solely determinative, factor in intervention efficacy. This pattern provides tentative evidence that incorporating game-like elements may enhance the efficacy of CCT, potentially by boosting intrinsic motivation, engagement, and emotional reward (Oldrati et al., 2020). However, the wide confidence interval for the low-gamification subgroup and the overall non-significant result indicate that this finding remains preliminary. Future studies specifically designed to directly compare gamified versus non-gamified protocols are warranted to confirm this potential added benefit.

Further subgroup analyses reinforced the robustness of the primary finding. The positive effect of CCT remained consistent across different intervention designs and samples with varying diagnostic compositions. Notably, the effect size was slightly larger when measured by the MoCA (g = 0.68) compared to the MMSE (g = 0.49). This is likely attributable to the MoCA’s superior sensitivity in assessing executive functions and detecting subtle cognitive changes, domains often targeted by CCT programs (Julayanont et al., 2012).

The considerable heterogeneity observed is a common feature in meta-analyses of complex interventions and likely originates from variations in training protocols, software platforms, and session parameters (Petropoulou et al., 2021; Terhorst et al., 2024). Furthermore, the wide 95% prediction interval suggests that the effect in a future application could range from negligible to large. This uncertainty underscores the importance of personalizing interventions and identifying patient-level moderators that may predict individual response.

### 4.2. Strengths and Limitations

To the best of our knowledge, this is the first meta-analysis to specifically isolate and evaluate the effect of task-based CCT delivered exclusively on tablet and computer platforms for individuals with MCI, and to systematically explore the degree of gamification as a potential effect modifier. This study adhered to rigorous methodological standards, including a comprehensive literature search, duplicate study selection and data extraction processes, utilization of the Cochrane RoB 2.0 tool, and pre-specified analyses employing robust statistical methods.

Several limitations must be acknowledged. First, the number of included studies, particularly within certain subgroups (e.g., low-gamification), was limited, which constrained the statistical power of the subgroup analyses. Second, the substantial clinical and methodological heterogeneity, although accounted for by a random-effects model, complicates the interpretation of a single pooled effect estimate. Third, regarding methodological quality, a significant proportion of studies were assessed as having ‘Some Concerns,’ primarily due to deviations from intended interventions. This reflects the inherent challenge of blinding in behavioral trials. Furthermore, factors specific to digital interventions, such as digital fatigue, device familiarity, and dropout rates (which led to ‘High Risk’ ratings in two studies), represent potential sources of bias that standard analyses may not fully mitigate. Fourth, the categorization of gamification degree, while based on a rubric, remains constrained by the absence of a universally standardized scale for quantifying gamification. Fifth, a notable limitation is the absence of reported negative effects or adverse events (e.g., eye strain, frustration) in the included studies. This lack of reporting may reflect a ‘file drawer’ problem regarding safety data in behavioral interventions, potentially leading to an underestimation of the burden on participants. Finally, the analysis focused solely on global cognition as the primary outcome; the effects on specific cognitive domains remain important avenues for future research.

### 4.3. Implications for Clinical Practice and Future Research

The findings provide robust evidence supporting the integration of tablet- and computer-based CCT as a feasible and effective complementary intervention into comprehensive management strategies for individuals with MCI.

The observed trend suggesting enhanced efficacy with higher degrees of gamification offers a practical insight for intervention designers. Incorporating game-like elements may represent a promising strategy for maximizing user engagement and adherence, which are critical for achieving sustained cognitive benefits. However, this approach should be balanced with usability considerations, particularly for older adults with varying levels of digital literacy.

Future research should prioritize several key areas: (1) conducting large-scale RCTs that directly compare gamified versus non-gamified CCT to definitively establish its additive value; (2) investigating the active components and optimal dosing parameters of effective training; (3) employing longer follow-up periods to assess the long-term sustainability of cognitive benefits and transfer to daily functioning; and (4) exploring personalized intervention strategies based on individual patient characteristics.

## 5. Conclusions

This meta-analysis demonstrates that computerized cognitive training administered through tablet and computer platforms effectively enhances global cognition in patients with mild cognitive impairment. While these widely accessible technologies provide a practical basis for intervention implementation, our findings underscore the complementary role of gamification in augmenting treatment outcomes. The consistent trend of increasing effect sizes with higher gamification levels indicates that well-designed game elements may significantly potentiate intervention efficacy, potentially through enhanced engagement and motivation. Future development should focus not only on leveraging the accessibility of these platforms but, more importantly, on systematically integrating and optimizing gamification principles to maximize both adherence and cognitive benefits.

Future development should focus not only on leveraging the accessibility of these platforms but, more importantly, on systematically integrating and optimizing gamification principles. However, it is important to note that unexplained clinical variability remains, which may be partly attributable to the use of broad screening instruments (e.g., MoCA) rather than specific neuropsychological batteries. To clarify these diverse effects and minimize methodological biases, future research must adopt standardized reporting protocols and rigorous study designs.

## Figures and Tables

**Table 1:** Characteristics of studies included in the systematic review.

Author, Year	Area	Age (Mean ± SD or Description)	Total Participants (Analyzed)	Patient Population	Outcomes Scale	Intervention Method	Intervention Device	Total Length	Frequency (/Week)	Session Length (min)
(Barnes et al., 2009)	USA	IG: 74.1 ± 8.7; CG: 74.8 ± 7.2	47 (IG: 22, CG: 25)	MCI	RBANS	Computerized Cognitive Training (Posit Science)	PC	6	5	100
(Hagovská & Olekszyová, 2016)	Slovakia	Overall mean: 67.07	80 randomized (IG: 40, CG: 40)	MCI	MMSE	CCT + Balance Training (CogniPlus)	PC	10	2	30
(Savulich et al., 2017)	United Kingdom	IG: 75.2 ± 7.4; CG: 76.9 ± 8.3	42 (IG: 21, CG: 21)	Amnestic MCI	MMSE	Game Show Training	Tablet	4	2	60
(Djabelkhir et al., 2017)	France	CCE: 78.2 ± 7.0; CCS: 75.2 ± 6.4	19 analyzed (randomized: 20)	MCI	MMSE	Computerized Cognitive Stimulation	Tablet	12	1	90
(Han et al., 2017)	Korea	74.01 ± 5.53	50 randomized	MCI	MMSE	uSMART (Spatial Reconstruction Task)	Tablet	4	2	30
(Frain & Chen, 2018)	USA	IG: 58 ± 6.5; CG: 54 ± 3.0	22 (IG: 10, CG: 12)	HIV-associated MCI	MoCA	Computerized Cognitive Training (BrainHQ)	PC	8	3	30
(Yang et al., 2019)	Taiwan	VIMT: 75.4 ± 6.6; PIA: 81.7 ± 7.2;	66 (VIMT: 33, PIA: 33)	MCI	MoCA	Computerized Cognitive Training (CogniPlus)	PC	12	3	45
(Bernini et al., 2019)	Italy	G1: 71.18; G2: 69.33	35 analyzed (G1: 17, G2: 18)	Parkinson’s Disease with MCI	MoCA	Computerized Cognitive Training	PC	4	3	45
(Weng et al., 2019)	China Mainland	/	62 analyzed (CCT: 33, MLA: 29)	MCI	MoCA	Working Memory CCT	PC	8	2	40–60
a (Bernini et al., 2021)	Italy	CCT: 74.61 ± 5.68; PCT: 69.83 ± 9.66;	35(CCT: 21 *; PCT: 14)	Parkinson’s Disease with MCI	MoCA	Computerized Cognitive Training (CoRe)	PC	3	4	45
b (Bernini et al., 2021)	Italy	CCT: 74.61 ± 5.68; CG: 69.33 ± 7.72	39 (CCT: 21 *; CG: 18)	Parkinson’s Disease with MCI	MoCA	Computerized Cognitive Training (CoRe)	PC	3	4	45
(Yeh et al., 2022)	Taiwan	SEQ: 53.05 ± 14.53; COG: 60.17 ± 12.13	56 (SEQ: 20, COG: 18)	Post-stroke MCI	MoCA	Aerobic Exercise, CCT, Sequential Combination	Tablet	12	3	60
(Duff et al., 2022)	USA	74.9	113 (IG: 55, CG: 58)	Amnestic MCI	RBANS	Computerized Cognitive Training (BrainHQ)	PC	12–13	4–5	45
(Lim et al., 2023)	Republic of Korea	IG: 75.42 ± 5.74; CG: 73.33 ± 17.52	24 (IG: 12, CG: 12)	MCI	MMSE	Serious Game (Brain Talk™)	Tablet	4	3	30
(Wu et al., 2023)	China Mainland	IG: 67.68 ± 5.83; CG: 65.52 ± 5.55	50 analyzed (IG: 25, CG: 25)	MCI	MoCA	Computerized Cognitive Training	PC	8	3	60
(Baik et al., 2024)	Republic of Korea	IG: 67.08 ± 7.92; CG: 65.64 ± 8.54	50 (IG: 25, CG: 25)	MCI	MoCA	Home-Based Computerized Cognitive Training (HB-CCT)	Tablet	8	3	30
(Graessel et al., 2024)	Germany	IG: 73.4 ± 8.1; CG: 73.5 ± 6.5	89 randomized (IG: 44, CG: 45)	MCI	MoCA	Individualized CCT (vs. basic CCT)	Tablet	24	3	30–35
(Wen et al., 2024)	China Mainland	IG: 66.78 ± 8.30; CG: 65.75 ± 9.41	118 randomized (IG: 37, CG: 81)	MCI	MoCA	CCT + Occupational Therapy	PC	12	3	≥30
(Petri et al., 2025)	Greece	IG: 82.5; CG: 80.9	20	MCI	MMSE	Computerized Cognitive Rehabilitation (RehaCom)	PC	6	2	50–60
(Ferizaj et al., 2025)	Germany	IG: 58.1 ± 12.9; CG: 59.6 ± 13.0	50 (IG: 36, CG: 14)	MCI	S-NAB	Computerized Cognitive Training	Tablet	12	3	25–40

Note: a, b: Indicate different comparisons (or intervention arms) from the same study. *: Indicates a shared group of participants used in multiple comparisons.

## Data Availability

No new data were created or analyzed in this study. Data sharing is not applicable to this article as it is a systematic review and meta-analysis of existing literature. The data presented in this study, including the extraction sheets and the R code used for meta-analysis, are available in the Supplementary Materials.

## Supplementary Materials

The following supporting information can be downloaded at: https://www.mdpi.com/article/10.3390/bs16010040/s1, Table S1: Reconciliation of included studies: Systematic review versus meta-analysis data availability; Table S2: Individual study results (Pre/Post data), effect sizes, and weights.
