# Supplementary material for: The Effects of Computerized Cognitive Training via Tablet and Computer Platforms on Cognitive Function in Patients with Mild Cognitive Impairment: A Systematic Review and Meta-Analysis
Source: Behav Sci (Basel). 2025 Dec 24;16(1):40. doi: 10.3390/bs16010040 (PMC12837966; doi:10.3390/bs16010040)
Supplement: Supplementary file 1 [file behavsci-16-00040-s001.zip › table S1.pdf]

Supplementary Table S1. Reconciliation of included studies: Systematic review versus meta-analysis data availability.

Study ID	Country	Included in SR?	Included in MA?	Reason for Exclusion / Notes
Baik et al., 2023	Republic of Korea	Yes	Yes	N/A
Barnes et al., 2009	USA	Yes	Yes	N/A
Bernini et al., 2019	Italy	Yes	Yes	N/A
Bernini et al., 2020 (Comparison a)	Italy	Yes	Yes	Comparison 1: CCT vs. Active Control
Bernini et al., 2020 (Comparison b)	Italy	Yes	Yes	Comparison 2: CCT vs. Passive Control
Djabelkhir et al., 2017	France	Yes	Yes	N/A
Duff et al., 2022	USA	Yes	Yes	N/A
Ferizaj et al., 2025	Germany	Yes	Yes	N/A
Frain & Chen, 2018	USA	Yes	Yes	N/A
Graessel et al., 2024	Germany	Yes	Yes	N/A
Hagovská et al., 2016	Slovakia	Yes	Yes	N/A
Han et al., 2017	Republic of Korea	Yes	Yes	N/A
Lim et al., 2023	Republic of Korea	Yes	Yes	N/A
Petri et al., 2025	Greece	Yes	Yes	N/A
Savulich et al., 2017	UK	Yes	Yes	N/A
Wen et al., 2024	China	Yes	Yes	N/A
Weng et al., 2019	China	Yes	Yes	N/A
Wu et al., 2023	China	Yes	Yes	N/A
Yang et al., 2019	Taiwan	Yes	Yes	N/A
Yeh et al., 2022	Taiwan	Yes	Yes	N/A
Total	19 Studies	19 Studies	20 Comparisons in Meta-analysis	
Excluded Study:				
Ma et al., 2024	*China*	*Excluded due to ineligible population (Depression without MCI)*		
