# Supplementary material for: The Effects of Computerized Cognitive Training via Tablet and Computer Platforms on Cognitive Function in Patients with Mild Cognitive Impairment: A Systematic Review and Meta-Analysis
Source: Behav Sci (Basel). 2025 Dec 24;16(1):40. doi: 10.3390/bs16010040 (PMC12837966; doi:10.3390/bs16010040)
Supplement: Supplementary file 1 [file behavsci-16-00040-s001.zip › table S2.pdf]

Supplementary Table S2. Individual study results (Pre/Post data), effect sizes, and weights.

**Study ID**	**Group**	**N**	**Baseline (Pre) Mean (SD)**	**Post-test Mean (SD)**	**Hedges' g [95% CI]**	**Weight (%)**
Duff et al.,	Int.	55	88.10 (12.50)	89.00 (13.10)	-0.20 [-0.57; 0.17]	7.10%
2022	Ctrl.	58	90.80 (12.90)	94.30 (12.10)		
Yang et al.,	Int.	33	24.64 (4.09)	25.85 (3.46)	0.07 [-0.42; 0.55]	6.10%
2019	Ctrl.	33	23.24 (3.95)	24.18 (4.30)		
Weng et al.,	Int.	33	17.45 (4.65)	18.09 (4.71)	0.29 [-0.22; 0.79]	6.00%
2019	Ctrl.	29	18.41 (3.40)	17.86 (3.32)		
Han et al.,	Int.	20	25.49 (3.40)	26.37 (2.99)	0.29 [-0.31; 0.90]	5.10%
2017	Ctrl.	22	25.83 (2.92)	25.76 (3.28)		
Barnes et	Int.	22	85.20 (11.50)	90.60 (11.20)	0.39 [-0.19; 0.97]	5.40%
al., 2009	Ctrl.	25	87.80 (13.60)	88.20 (13.30)		
Graessel et	Int.	44	22.00 (1.80)	24.00 (2.90)	0.43 [0.01; 0.85]	6.60%
al., 2024	Ctrl.	45	22.30 (1.50)	23.30 (2.40)		
Hagovská et	Int.	40	25.97 (2.57)	26.97 (2.21)	0.45 [0.00; 0.90]	6.40%
al., 2016	Ctrl.	38	26.02 (1.47)	26.10 (1.46)		
Ferizaj et	Int.	36	93.50 (16.40)	99.30 (16.40)	0.48 [-0.14; 1.11]	5.00%
al., 2025	Ctrl.	14	92.10 (15.30)	90.10 (13.00)		
Djabelkhir	Int.	9	27.00 (2.00)	28.00 (1.40)	0.49 [-0.43; 1.41]	3.30%
et al., 2017	Ctrl.	10	27.70 (1.90)	27.80 (1.50)		
Savulich et	Int.	21	26.60 (2.90)	27.40 (3.97)	0.50 [-0.11; 1.12]	5.10%
al., 2017	Ctrl.	21	26.80 (2.20)	26.00 (2.94)		
Bernini et	Int.	18	19.09 (2.84)	21.45 (2.60)		
al., 2020*(b)*	Ctrl.	12	20.93 (3.42)	21.50 (3.39)	0.55 [-0.34; 1.43]	3.50%
al., 2020*(a)*	Ctrl.	18	19.17 (3.49)	19.11 (3.32)	0.73 [-0.10; 1.56]	3.80%
Yeh et al.,	Int.	20	19.15 (4.90)	24.25 (4.49)	0.55 [-0.10; 1.20]	4.90%
2022	Ctrl.	18	19.39 (4.33)	21.72 (5.64)		
Lim et al.,	Int.	12	24.42 (1.98)	27.75 (1.54)	0.64 [-0.19; 1.46]	3.80%
2023	Ctrl.	12	23.83 (2.89)	25.50 (3.23)		
Frain &	Int.	10	22.00 (1.90)	24.10 (1.90)	0.66 [-0.20; 1.53]	3.60%
Chen, 2018	Ctrl.	12	21.30 (2.70)	21.80 (2.50)		
Bernini et	Int.	17	20.82 (3.34)	23.52 (2.78)	0.83 [0.13; 1.52]	4.60%
al., 2019	Ctrl.	18	19.17 (3.49)	19.11 (3.32)		
Wen et al.,	Int.	37	20.49 (2.56)	21.95 (3.06)	0.93 [0.52; 1.34]	6.80%
2024	Ctrl.	81	20.15 (3.82)	18.14 (4.20)		
Petri et al.,	Int.	10	26.40 (1.35)	27.70 (1.41)	1.06 [0.11; 2.01]	3.20%
2025	Ctrl.	10	25.50 (1.95)	25.00 (1.70)		
Wu et al.,	Int.	25	20.40 (2.42)	24.52 (2.80)	1.42 [0.79; 2.04]	5.00%
2023	Ctrl.	25	21.64 (2.27)	21.88 (3.06)		
Baik et al.,	Int.	25	21.12 (1.27)	23.84 (2.21)	1.68 [1.03; 2.33]	4.80%
2023	Ctrl.	25	20.84 (1.46)	20.52 (1.76)		
